# Regional Analysis of Liver Surface Nodularity in a Single Axial MR Image for Staging Liver Fibrosis

**DOI:** 10.1002/jmri.28208

**Published:** 2022-05-11

**Authors:** Tae‐Hoon Kim, Youe Ree Kim, Chang‐Won Jeong, Hyung Joong Kim, Jin Woong Kim, Young Hwan Lee, Kwon‐Ha Yoon

**Affiliations:** ^1^ Medical Convergence Research Center Wonkwang University Iksan Republic of Korea; ^2^ Department of Radiology Wonkwang University School of Medicine and Wonkwang University Hospital Iksan Republic of Korea; ^3^ Department of Biomedical Engineering Kyung Hee University Dongdaemun‐gu, Seoul Republic of Korea; ^4^ Department of Radiology Chosun University College of Medicine, Chosun University Hospital Gwangju Korea

**Keywords:** liver surface nodularity (LSN), regional analysis, liver fibrosis

## Abstract

**Background:**

The assessment of liver surface nodularity (LSN) for staging hepatic fibrosis is restricted in clinical practice because it requires customized software and time‐consuming procedures. A simplified method to estimate LSN score may be useful in the clinic.

**Purpose:**

To evaluate the regional analysis of LSN and processing time in a single axial liver MR image for staging liver fibrosis.

**Study Type:**

Retrospective.

**Population:**

A total of 210 subjects, a multicenter study.

**Field Strength/Sequence:**

A 3 T/noncontrast gradient echo T1WI.

**Assessment:**

Subjects were divided into five fibrosis groups (F_0_ = 29; F_1_ = 20; F_2_ = 32; F_3_ = 50; F_4_ = 79) based on the METAVIR fibrosis scoring system. The mean LSN (on three slices) and regional LSN (on one slice) measurements, and the processing times, are compared. The regional LSN scores in five regions‐of‐interests (ROI_1‐5_) were analyzed in a single axial MRI at the level of the hilum by two independent observers.

**Statistical Tests:**

Regional variations in LSN scores were compared using ANOVA with Tukey test. Agreement between the mean and regional LSN measurements was evaluated using Pearson correlation coefficients (*r*) and Bland–Altman plots. The diagnostic performance of mean and regional LSN scores according to fibrosis stage was evaluated with the AUROC. A *P* value < 0.05 was considered statistically significant.

**Results:**

Total processing time for a regional LSN measurement (3.6 min) was 75.5% less than that for mean LSN measurement (14.7 min). Mean LSN scores and all five regional LSN scores showed significant differences between fibrosis groups. Among regional LSN scores, ROI_5_ showed the highest AUROC (0.871 at cut‐off 1.12) for discriminating F_0‐2_ vs. F_3‐4_ and the best correlation with mean LSN score (*r* = 0.800, −0.07 limit of agreement).

**Conclusion:**

Quantitative regional LSN measurement in a single axial MR image reduces processing time. Regional ROI_5_ LSN score might be useful for clinical decision‐making and for distinguishing the difference between early fibrosis (F_0‐2_) and advanced fibrosis (F_3−4_) in the liver.

**Evidence Level:**

3

**Technical Efficacy:**

Stage 2

Noninvasive evaluation of liver fibrosis is challenging in clinical practice.^1^ In an initial diagnosis of early stage hepatic fibrosis (compensated liver) or cirrhosis (decompensated liver as end stage), it is difficult to accurately predict hepatic compensation or decompensation using noninvasive methods.^2^ Therefore, there is an unmet need for widely applicable noninvasive methods to diagnose fibrosis and advanced cirrhosis and to predict future risk of hepatic decompensation.

To diagnose fibrosis and cirrhosis of the liver, the importance of assessing liver surface nodularity (LSN) is emerging in the clinical setting.^3^, ^4^ The LSN score can be measured from routine liver CT and MR images using postprocessing software.^3^, ^4^, ^5^, ^6^ In clinical practice, a quantitative LSN score may be used to diagnose and stage various liver diseases including chronic liver disease (CLD) and nonalcoholic fatty liver disease (NAFLD). Smith et al and Pickhardt et al reported that the quantitative LSN score in CT images progressively increases with higher clinical stages of CLD and/or cirrhosis (end‐stage) and is predictive of cirrhosis decompensation and death.^4^, ^5^, ^6^ Recently, Kim et al., who developed a Matlab‐based LSN quantification software using multipolynomial curve‐fitting analysis,^3^ reported that the use of quantitative LSN software can help to differentiate the stage of liver fibrosis in NAFLD (fibrosis grade F_0‐1_ vs. F_2‐3_ based on pathologic NAFLD activity score) and in CLD (fibrosis grades F_0_‐F_3_ based on serum fibrosis‐4 index).^3^, ^7^ The LSN score has high reproducibility on each CT^4^, ^6^ and MRI^7^, ^8^ study for staging liver fibrosis and end‐stage cirrhosis. However, the mean LSN scores reported in MRI studies^7^, ^8^ were different from CT studies^4^, ^6^ because they used a different software with a different methodology. Moreover, it has restricted use in clinical practice because it requires customized software for quantification and the LSN assessment of the whole liver is a time‐consuming procedure. Therefore, the assessment of LSN scores using a simplified method, such as measurement from a single image, may be useful.

Thus, the purpose of this study was to compare the regional LSN scores and processing times derived from a single slice (with mean LSN score derived from the whole liver) for assessing liver fibrosis on a single axial liver MR image and to evaluate the diagnostic performance of LSN score in hepatic fibrosis.

## Materials and Methods

The retrospective study was approved by our institutional review board (No. 2018‐01‐005) and the requirement for written informed consent was waived.

### Subject Population

Consecutive patients from January 2010 to June 2020, who were over 20 years of age, who underwent abdominal MRI and who had available pathologic information and serologic tests were retrospectively identified at four medical centers. A total of 210 patients who had undergone 3.0 T liver MRI before or after serum biochemistry and histopathologic investigation were enrolled (*n* = 85 from Wonkwang University Hospital, *n* = 82 from Chonbuk National University Hospital, *n* = 14 from Asan Medical Center and *n* = 29 from Chonnam National University Hospital). This study excluded patients (*n* = 9) who had undergone hepatectomy or had a pathologic condition including a large bulging mass that could interfere with measurement of LSN on the anterior or anterolateral surfaces of the liver.^8^ Also, we excluded MR images (*n* = 2) impaired by artifacts that can affect image quality and lead to loss of diagnostic information.^9^

### Reference Standard for Liver Fibrosis

**FIGURE 1:**
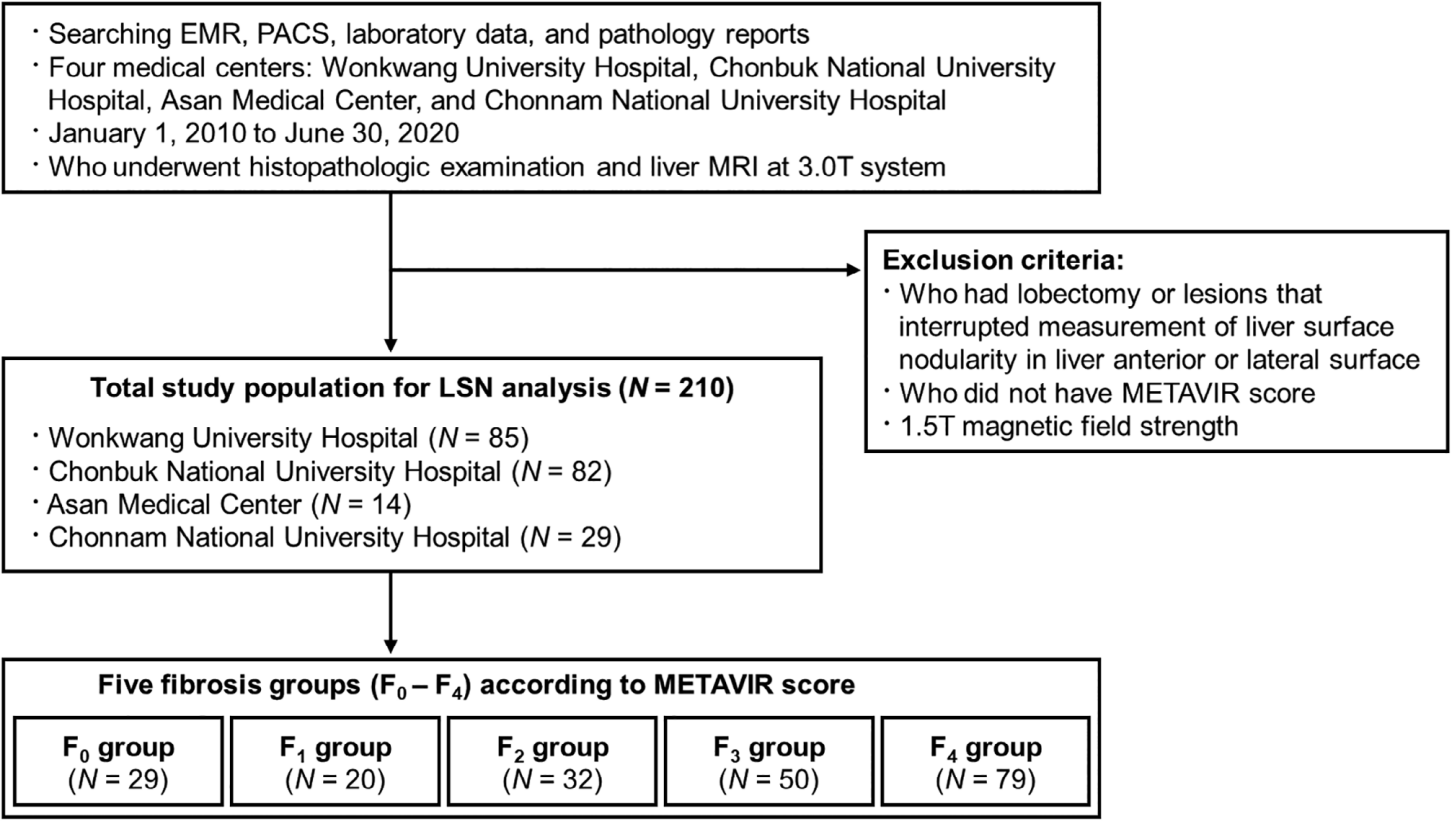
Flowchart for inclusion of study population. EMR = electronic medical records; LSN = liver surface nodularity

All histopathological information was obtained by percutaneous needle biopsy or surgical biopsy. The histological data were analyzed by a pathologist with 9‐years' experience based on the Meta‐analysis of Histological Data in Viral Hepatitis (METAVIR) fibrosis scoring system,^10^, ^11^ as follows: F_0_, no fibrosis; F_1_, portal fibrosis; F_2_, periportal fibrosis; F_3_, septal fibrosis; and F_4_, cirrhosis. The final cohort was divided into five groups (F_0_‐F_4_) according to hepatic fibrosis stages as follows: F_0_ (*n* = 29), F_1_ (*n* = 20), F_2_ (*n* = 32), F_3_ (*n* = 50), and F_4_ (cirrhosis, *n* = 79) (Fig. 1). The patients with F_0_‐F_2_ scores and F_3_‐F_4_ scores were classified as early fibrosis and advanced fibrosis, respectively.

### Acquisition of MR Images

All imaging studies were performed on MR scanners with body matrix coils. Unenhanced fat‐suppressed T1‐weighted 3D spoiled gradient‐echo images in axial plane were acquired with following parameters: TR/TE = 4.2/1.97 msec, field of view = 38 × 38 × 14 cm^3^, matrix size = 512 × 512, number of excitations = 2, slice thickness = 2.0 mm, and number of slices = 70.

A total of four 3.0 T MRI scanners were used. Two MR scanners (Achieva [*n* = 40]; Ingenia CX [*n* = 45], Philips Healthcare, Best, The Netherlands) were used at Wonkwang University Hospital. At Chonbuk National University Hospital, three MR scanners (Verio [*n* = 46]; Skyra [*n* = 13], Siemens healthineers, Erlangen, Germany) (Achieva, Philips Healthcare [*n* = 23]) were used. At Asan Medical Center, two MR scanners (Skyra, Siemens [*n* = 13]; Ingenia CX, Philips [*n* = 1]) were used. At Chonnam National University Hospital, a scanner (Skyra, Siemens [*n* = 29]) scanner was used.

### Preprocessing and Quantification of MR Data for LSN Assessment on Single Slice

The LSN quantification software was developed in Matlab (R2018a; MathWorks, Natick, Massachusetts).^3^ The Wonkwang Abdomen and Liver Total Solution (WALTS) is a customized postprocessing program that operates on the Windows platform (ver. XP or higher; Microsoft, Redmond, WA). This was used to measure LSN on the DICOM format images to derive an LSN score with the same procedure as in prior studies.^3^, ^7^, ^8^ The main algorithm for assessing the LSN was as follows: bias field correction of images via the level set method, semi‐automatic liver boundary detection for drawing the liver reference line, liver segmentation, and LSN measurements with multipolynomial curve‐fitting analysis.

### LSN Analysis in Clinical Patients

**FIGURE 2:**
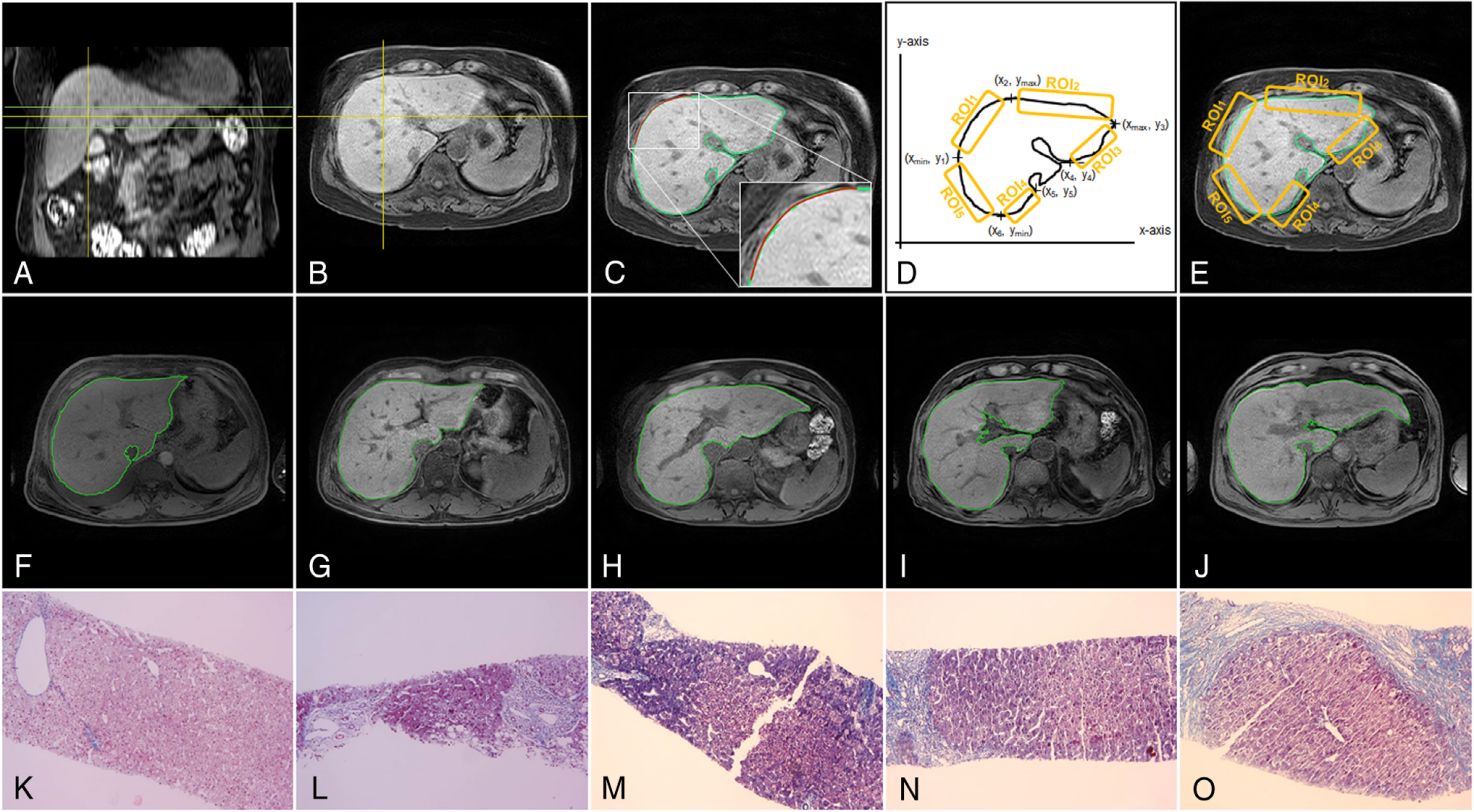
Representative postprocessing procedures for quantification of liver surface nodularity (LSN) (a–e), MR images (f–j) and pathologic images (Masson's trichrome stain, original magnification ×40) (k–o) in each fibrosis stage. (a) Three axial slices (horizontal lines) selected at the level of the hepatic hilum. (b) Mean LSN score in each subject calculated from three slices (vertical line shows location of sagittal sectional image in a). (c) Final liver surface line (green line) and LSN measurement (curve‐fitting line = red line). (d) Schematic drawing for selection of regions‐of‐interests (ROIs) from six datapoints. (e) Regional LSN scores calculated from five different ROIs on a single axial MR image. (f, k) F_0_ (57 year old, F), (g, l) F_1_ (30 year old, M), (h, m) F_2_ (64 year old, F), (i, n) F_3_ (72 year old, M), and (j, o) F_4_ (52 year old, M), respectively.

All MR images were reviewed on standard PACS stations with standard window settings. LSN analysis using customized WALTS software was independently performed by two radiologists (abdominal radiologists with 9‐years [Y.R.K] and 29‐years' [K.H.Y] experience, respectively, in liver imaging) blinded to the radiologic reports of MR datasets. Each radiologist selected three image slices at the level of the hepatic hilum for quantitative analysis (Fig. 2).^7^, ^12^, ^13^ Then, bias correction and liver segmentation were performed on the selected slices. LSN is measured on a pixel‐by‐pixel basis and measures the mean distance for each line (expressed as tenths of a millimeter). The final LSN score (mean LSN score and regional LSN score) is the arithmetic mean of the measurements as following procedures.

To measure mean LSN score as a reference value, the mean LSN score in an individual subject was calculated as an averaged value measured from three slice images at the level of the hilum (vertical lines as shown in Fig. 2a). LSN score on every single slice (Fig. 2b) estimated from five regions‐of‐interests (ROIs) (Fig. 2d,e).

To measure regional LSN, five ROIs (ROI_1‐5_) on one of three slices were analyzed to measure the regional LSN scores with a fourth‐degree polynomial curve‐fitting (Fig. 2c). The data points for the selection of ROIs on the segmented liver image are shown in Fig. 2d,e. The datapoints are on the basis of the bottom‐left corner origin position (*x*, *y* coordinate = 0, 0) in a single axial image (Fig. 2d).

All selected MR images were analyzed using both mean (from three slices) and regional (from one slice) LSN measurements. To determine interobserver agreement, two radiologists were independently measured LSN score on selected images by each method. Details of the LSN measurement technique for clinical patients are described in recent studies.^3^, ^7^ Following preprocessing of MR image data, liver parenchyma within the liver surface line was used for the deterministic curve‐fitting analysis. ROIs were selected along the boundary of the liver and a smooth curve‐fitting line (fourth‐order line shape) was generated on the selected ROIs. Finally, the difference between the liver surface line (green line, Fig. 2c) and the new curve‐fitting line (red line, Fig. 2c) was measured on a pixel‐by‐pixel basis. The difference value was squared, and the mean value, variation, and standard deviation (SD) were calculated. The overall LSN scores in individual subjects were calculated as the arithmetic mean and regional scores of the measurements, with a higher LSN score indicating more hepatic surface nodularity. The processing time for comparing the mean and regional LSN score was recorded within the WALTS program.

### Statistical Analysis

Differences in mean and regional LSN scores between fibrosis groups (F_0_‐F_4_) as well as serum biochemistry were analyzed using one‐way analysis of variance (ANOVA) with Tukey's post hoc test using the statistical package for the social sciences program (SPSS ver. 20; Chicago, IL, USA). The coefficient of variance (CV) in each ROI group was calculated to assess the variability of measurements.^14^ Pearson correlation between the five regional LSN measurements and the mean LSN scores was performed. Agreement between the LSN measurements of the two methods was evaluated by calculating Pearson correlation coefficients (*r*) and generating Bland–Altman plots. Pearson correlation and Bland–Altman analysis were performed using MedCalc ver.14.8.1 (Medcalc, Mariakerke, Belgium).^15^ Intraclass correlation coefficients (ICC) values were evaluated to assess interobserver agreement in LSN scores and were classified as^16^: poor (<0.40), moderate (0.40 to <0.60), good (0.60 to <0.80), and excellent (0.80–1.00).

To evaluate the diagnostic performance of mean and regional LSN scores according to fibrosis stages, receiver operating characteristics (ROC) curve analysis was performed to calculate the area under the ROC curve (AUROC), sensitivity, specificity, and diagnostic accuracy (DA). Two‐sided *P* values less than 0.05 were considered to indicate statistical significance.

## Results

### Patient Characteristics

The etiology and serum biochemistry of the enrolled 210 patients (148 men, 62 women; mean age, 56.2 ± 13.2 years) are shown in Table 1. These patients had various liver diseases, including chronic hepatitis B (*n* = 110), chronic hepatitis C (*n* = 9), coinfection (*n* = 4), alcoholic liver disease (*n* = 18), NAFLD/steatohepatitis (NASH) (*n* = 18), and autoimmune hepatitis (*n* = 13). Thirty‐eight patients had liver disease of unknown cause. The results of blood chemistry showed significant differences between fibrosis stages in albumin, alanine aminotransferase, aspartate aminotransferase, and platelet count.

**TABLE 1:** Etiology of Study Population and Clinical Data of Serum Biochemistry in METAVIR Fibrosis Stages (F)

	Total (*n* = 210)	F_0_ (*n* = 29)	F_1_ (*n* = 20)	F_2_ (*n* = 32)	F_3_ (*n* = 50)	F_4_ (*n* = 79)	*P* value* ^,^ ^†^
Age (year)	56.2 ± 13.2	46.8 ± 20.9	54.4 ± 16.9	57.5 ± 10.9	59.7 ± 10.5	57.4 ± 9.0	<0.001^bcd,^ ^†^
Male: Female	148:62	21:8	13:7	21:11	3515	58:21	0.904*
Etiology*							<0.001*
Hepatitis B	110	2	9	14	35	50	
Hepatitis C	9	1	0	3	3	2	
Coinfection	4	0	0	0	2	2	
Alcohol	18	1	1	2	3	11	
NAFLD	18	1	6	5	0	6	
Autoimmune	13	0	1	4	4	4	
Unknown cause	38	24	3	4	3	4	
Serum biochemistry^†^							
Albumin (g/dL)	4.14 ± 0.03	4.35 ± 0.05	4.12 ± 0.08	4.05 ± 0.06	4.27 ± 0.05	3.98 ± 0.07	^†^0.003^dj^
ALT (IU/L)	34.6 ± 1.78	20.56 ± 1.55	47.94 ± 12.54	35.73 ± 3.30	34.76 ± 3.23	36.92 ± 2.26	^†^0.011^a^
AST (IU/L)	41.5 ± 2.65	24.26 ± 1.65	57.13 ± 21.28	44.95 ± 7.69	37.31 ± 2.17	47.56 ± 2.83	0.036^a^ ^†^
Platelet count (×10^3^/μL)	170.9 ± 4.73	221.30 ± 17.70	175.56 ± 14.48	165.04 ± 8.41	173.93 ± 6.92	143.69 ± 7.12	<0.001^bcd,^ ^†^

ALP = alkaline phosphatase; ALT = alanine aminotransferase; AST = aspartate aminotransferase; NAFLD = non‐alcoholic fatty liver disease.

Etiology data are presented as the number of patients. The value in parenthesis indicates the percentage as the number of patients/total number of patients × 100.

Serum biochemistry data are presented as mean ± SEM. Serum levels in the healthy control group are used as reference ranges.

* The difference among fibrosis groups in etiology data was analyzed by Pearson's chi‐square test.

^†^ The difference among three fibrosis groups was analyzed by one‐way ANOVA with Tukey post hoc test as follows: ^a^F_0_ vs. F_1_, ^b^F_0_ vs. F_2_, ^c^F_0_ vs. F_3_, ^d^F_0_ vs. F_4_, ^e^F_1_ vs. F_2_, ^f^F_1_ vs. F_3_, ^g^F_1_ vs. F_4_, ^h^F_2_ vs. F_3_, ^i^F_2_ vs. F_4_, and ^j^F_3_ vs. F_4_.

### LSN Measurements in Different ROIs

Figure 2 shows representative postprocessing procedures (a–e), MR images (f–j), and histopathologic images (k–o). The processing time and LSN scores measured from two observers present an averaged value. The median time for quantifying mean LSN measurement from three image slices was 5.1 minutes (range, 2.1–6.0 minutes). For quantifying a single ROI LSN score, it was 0.4 minutes (range, 0.3–0.6 minutes) (92.2% time reduction). The median time for additional postprocessing (including bias correction, boundary detection, and liver surface line drawing) was 3.2 minutes (range, 2.4–5.4 minutes) per single image. The total processing time was 3.6 minutes (range, 2.7–6.0 minutes) for the single ROI measurement method and 14.7 minutes (range, 9.5–22.2 minutes) for the mean LSN measurement method.

Mean and regional LSN scores in ROIs were significantly different in fibrosis stages (Table 2). The CV value of mean LSN scores was 6.2% (range 5%–8%) and the averaged CV values in each ROI were 15.4% for ROI_1_, 17.6% for ROI_2_, 18.4% for ROI_3_, 13.6% for ROI_4_, and 13.2% for ROI_5_ (range 11–24%; average 17.3%). The mean LSN scores in each fibrosis stage were significantly different for all fibrosis stage comparisons. Regional LSN scores were also significantly different for most fibrosis stage comparisons but not for F_0_ vs. F_1_ (ROI_1_ *P* = 0.493; ROI_2_ *P* = 0.082; ROI_3_ *P* = 0.959; ROI_4_ *P* = 0.996; ROI_5_ *P* = 0.516), F_2_ vs. F_3_ (ROI_1_ *P* = 0.485; ROI_2_ *P* = 0.283; ROI_3_ *P* = 0.561; ROI_4_ *P* = 0.539), and F_3_ vs. F_4_ (ROI_1_ *P* = 0.072; ROI_3_ *P* = 0.144; ROI_4_ *P* = 0.112; ROI_5_ *P* = 0.205).

**TABLE 2:** Comparison of LSN Scores in Different Region of Interests According to Fibrosis Stages

	ROI_1_ LSN	ROI_2_ LSN	ROI_3_ LSN	ROI_4_ LSN	ROI_5_ LSN	Mean LSN
F_0_ (rank)	0.93 ± 0.14 [15] (1)	0.49 ± 0.12 [24] (5)	0.73 ± 0.16 [21] (2)	0.78 ± 0.14 [18] (4)	0.80 ± 0.08 [11] (2)	0.75 ± 0.04 [5]
F_1_	1.04 ± 0.12 [12] (1)	0.61 ± 0.08 [13] (5)	0.79 ± 0.09 [12] (3)	0.80 ± 0.10 [13] (4)	0.89 ± 0.11 [12] (2)	0.82 ± 0.05 [6]
F_2_	1.26 ± 0.17 [14] (1)	0.82 ± 0.13 [15] (5)	1.09 ± 0.16 [15] (3)	1.08 ± 0.19 [18] (4)	1.14 ± 0.18 [16] (2)	1.08 ± 0.06 [5]
F_3_	1.35 ± 0.27 [20] (1)	0.90 ± 0.19 [21] (5)	1.21 ± 0.27 [23] (3)	1.14 ± 0.22 [19] (4)	1.26 ± 0.18 [14] (2)	1.17 ± 0.10 [8]
F_4_	1.44 ± 0.22 [16] (1)	0.99 ± 0.15 [15] (5)	1.30 ± 0.27 [21] (3)	1.23 ± 0.22 [18] (4)	1.33 ± 0.18 [13] (2)	1.26 ± 0.09 [7]
*P* value*	<0.001	<0.001	<0.001	<0.001	<0.001	<0.001
F_0_ vs. F_1_	0.364	0.050	0.906	0.998	0.348	0.005
F_0_ vs. F_2_	<0.001	<0.001	<0.001	<0.001	<0.001	<0.001
F_0_ vs. F_3_	<0.001	<0.001	<0.001	<0.001	<0.001	<0.001
F_0_ vs. F_4_	<0.001	<0.001	<0.001	<0.001	<0.001	<0.001
F_1_ vs. F_2_	0.003	<0.001	<0.001	<0.001	<0.001	<0.001
F_1_ vs. F_3_	<0.001	<0.001	<0.001	<0.001	<0.001	<0.001
F_1_ vs. F_4_	<0.001	<0.001	<0.001	<0.001	<0.001	<0.001
F_2_ vs. F_3_	0.388	0.167	0.122	0.618	0.012	<0.001
F_2_ vs. F_4_	0.001	<0.001	<0.001	0.003	<0.001	<0.001
F_3_ vs. F_4_	0.148	0.004	0.299	0.095	0.195	<0.001

LSN = liver surface nodularity; ROI = region of interest.

Data presented mean ± SD (coefficient of variation [CV]). The values in parenthesis indicated the ranking of LSN score in each fibrosis stage.

* The difference among fibrosis groups within each ROI was analyzed by the one‐way ANOVA with Tukey's post hoc test.

### Correlations Between Mean and Regional LSN Measurement Methods

**FIGURE 3:**
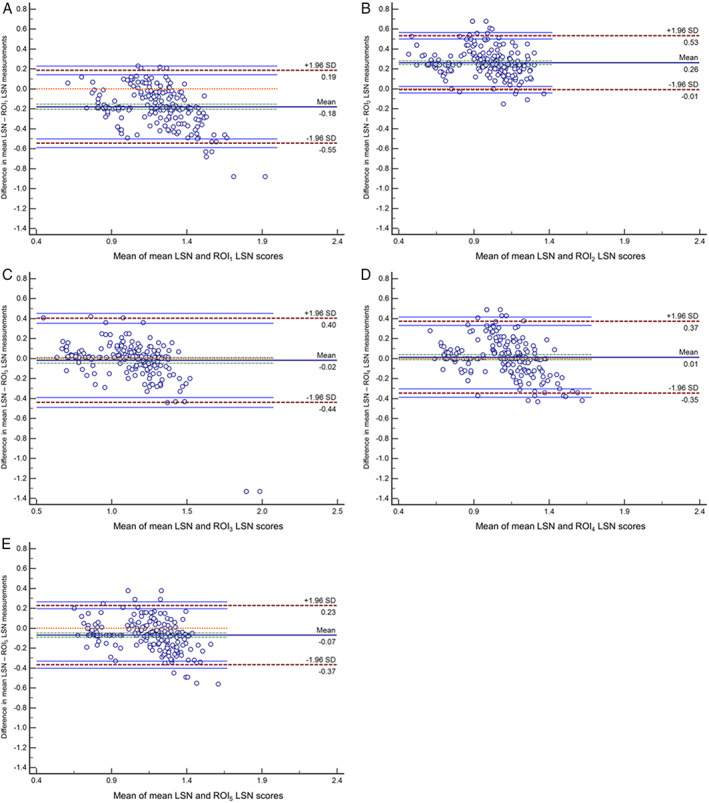
Bland–Altman liver surface nodularity (LSN) measurement agreement. Bland–Altman LSN measurement agreement plots between mean LSN and ROI measurement methods: (a) mean LSN vs. ROI_1_ LSN measurement; (b) mean LSN vs. ROI_2_ LSN measurement; (c) mean LSN vs. ROI_3_ LSN measurement; (d) mean LSN vs. ROI_4_ LSN measurement; and (e) mean LSN vs. ROI_5_ LSN measurement. The mean error is shown as the central dashed line, and the 95% limits of agreement (±1.96 standard deviation [SD]) are shown as the peripheral dashed lines. Note that *x*‐ and *y*‐axis scales different from each other.

Figure 3 shows the Bland–Altman measurement agreement plots between the two measurement methods. Table 3 shows the associations between the mean and each ROI_*n*_ LSN score. The systemic bias between mean and ROI_*n*_ LSN scores was −0.18 (limits of agreement [LOA] of −0.55 and 0.19) for ROI_1_, 0.26 (LOA of −0.01 and 0.53) for ROI_2_, −0.02 (LOA of −0.44 and 0.40) for ROI_3_, 0.01 (LOA of −0.35 and 0.37) for ROI_4_, and −0.07 (LOA of −0.37 and 0.23) for ROI_5_, respectively. There were significant correlations between mean and regional LSN measurements using the Pearson correlation coefficient (*r =* 0.740 for ROI_1_, *r =* 0.807 for ROI_2_, *r =* 0.732 for ROI_3_, *r =* 0.715 for ROI_4_, and *r =* 0.800 for ROI_5_). Among them, the regional ROI_5_ LSN score (*r* = 0.800) with −0.07 LOA had the best correlation with the mean LSN score.

**TABLE 3:** Correlation Between Mean LSN Score and Each ROI_*n*_ LSN

	ROI_1_	ROI_2_	ROI_3_	ROI_4_	ROI_5_
Pearson's coefficient (*r*)	0.740**	0.807**	0.732**	0.715**	0.800**
*P* value*	<0.001	<0.001	<0.001	<0.001	< 0.001

LSN = liver surface nodularity; ROI = region of interest.

The correlation coefficient was analyzed by the Pearson's test as following significance:*P < 0.05 and **P < 0.01.

### ROC Analysis for Differential Diagnosis According to Fibrosis Stages

The AUROC of LSN scores for the differentiation of fibrosis stages and their DA is summarized in Table 4. Mean LSN scores showed excellent diagnostic performance with the highest sensitivities and specificities (AUROC 0.994 [95% confidence interval 0.986–1.00 for F_0_‐F_1_ vs. F_2_‐F_4_; 0.946 [0.920–0.973] for F_0_‐F_2_ vs. F_3_‐F_4_; 0.884 [0.842–0.926] for F_0_‐F_3_ vs. F_4_). The AUROCs of regional LSN scores in differentiating early fibrosis (F_0_‐F_2_) from advanced fibrosis (F_3_‐F_4_) were 0.820 (0.764–0.876) for ROI_0_ LSN; 0.869 (0.823–0.915) for ROI_2_ LSN; 0.863 (0.810–0.916) for ROI_3_ LSN; 0.817 (0.760–0.874) for ROI_4_ LSN; and 0.871 (0.822–0.920) for ROI_5_ LSN. ROI_5_ LSN score showed the highest diagnostic performance for differentiating F_0_‐F_2_ from F_3_‐F_4_. DA (%, number of subject) for discriminating between early fibrosis and advanced fibrosis (F_0‐2_ vs. F_3‐4_) was 72.4% (152/210) for ROI_1_, 78.6% (165/210) for ROI_2_, 82.9% (174/210) for ROI_3_, 73.3% (154/210) for ROI_4_, and 81.9% (172/210) for ROI_5_. For mean LSN, the DA was 89%.

**TABLE 4:** Receiver Operator Curve (ROC) Analysis for Diagnosing Fibrosis Stage Using Regional LSN Scores

Comparison	Threshold Value	Sensitivity (%)	Specificity (%)	PPV (%)	NPV (%)	DA (%)	AUROC	*P* value
Mean LSN score								
F_0‐1_ (*n* = 49) vs. F_2‐4_ (*n* = 161)	0.93	98.8 (159/161)	98.0 (48/49)	99.4 (159/160)	96.0 (48/50)	98.6 (207/210)	0.999	<0.001
F_0‐2_ (*n* = 81) vs. F_3‐4_ (*n* = 129)	1.12	89.1 (115/129)	88.9 (72/81)	92.7 (115/124)	83.7 (72/86)	89.0 (187/210)	0.967	<0.001
F_0‐3_ (*n* = 131) vs. F_4_ (*n* = 79)	1.18	83.5 (66/79)	80.2 (105/131)	71.7 (66/92)	89.0 (105/118)	81.4 (171/210)	0.900	<0.001
ROI_1_ LSN score								
F_0‐1_ (*n* = 49) vs. F_2‐4_ (*n* = 161)	1.13	84.5 (136/161)	85.7 (42/49)	95.1 (136/143)	62.7 (42/67)	84.8 (178/210)	0.942	<0.001
F_0‐2_ (*n* = 81) vs. F_3‐4_ (*n* = 129)	1.24	72.1 (93/129)	72.8 (59/81)	80.9 (93/115)	62.1 (59/95)	72.4 (152/210)	0.837	<0.001
F_0‐3_ (*n* = 131) vs. F_4_ (*n* = 79)	1.31	69.6 (55/79)	68.7 (90/131)	57.3 (55/96)	78.9 (90/114)	69.0 (145/210)	0.774	<0.001
ROI_2_ LSN score								
F_0‐1_ (*n* = 49) vs. F_2‐4_ (*n* = 161)	0.67	90.7 (146/161)	89.8 (44/49)	96.7 (146/151)	74.6 (44/59)	90.5 (190/210)	0.970	<0.001
F_0‐2_ (*n* = 81) vs. F_3‐4_ (*n* = 129)	0.83	78.3 (101/129)	79.0 (64/81)	85.6 (101/118)	69.6 (64/92)	78.6 (165/210)	0.883	<0.001
F_0‐3_ (*n* = 131) vs. F_4_ (*n* = 79)	0.92	75.9 (60/79)	77.9 (102/131)	67.4 (60/89)	84.3 (102/121)	77.1 (162/210)	0.822	<0.001
ROI_3_ LSN score								
F_0‐1_ (*n* = 49) vs. F_2‐4_ (*n* = 161)	0.94	93.8 (151/161)	93.9 (46/49)	98.1 (151/154)	82.1 (46/56)	93.8 (197/210)	0.978	<0.001
F_0‐2_ (*n* = 81) vs. F_3‐4_ (*n* = 129)	1.09	82.9 (107/129)	82.7 (67/81)	88.4 (94/116)	75.3 (67/89)	82.9 (174/210)	0.886	<0.001
F_0‐3_ (*n* = 131) vs. F_4_ (*n* = 79)	1.16	69.6 (55/79)	68.7 (90/131)	57.3 (55/96)	78.9 (90/114)	69.0 (145/210)	0.792	<0.001
ROI_4_ LSN score								
F_0‐1_ (*n* = 49) vs. F_2‐4_ (*n* = 161)	0.93	86.3 (139/161)	85.7 (42/49)	95.2 (139/146)	65.6 (42/64)	86.2 (181/210)	0.935	<0.001
F_0‐2_ (*n* = 81) vs. F_3‐4_ (*n =* 129)	1.06	72.9 (94/129)	74.1 (60/81)	81.7 (94/115)	63.2 (60/95)	73.3 (154/210)	0.834	<0.001
F_0‐3_ (*n* = 131) vs. F_4_ (*n* = 79)	1.13	69.6 (55/79)	70.2 (92/131)	58.5 (55/94)	79.3 (92/116)	70.0 (147/210)	0.763	<0.001
*ROI*_*5*_ *LSN score*								
F_0‐1_ (*n* = 49) vs. F_2‐4_ (*n* = 161)	1.01	94.4 (152/161)	93.9 (46/49)	98.1 (152/155)	83.6 (46/55)	94.3 (198/210)	0.977	<0.001
F_0‐2_ (*n* = 81) vs. F_3‐4_ (*n* = 129)	1.13	82.2 (106/129)	81.5 (66/81)	87.6 (106/121)	74.2 (66/89)	81.9 (172/210)	0.889	<0.001
F_0‐3_ (*n* = 131) vs. F_4_ (*n* = 79)	1.24	73.4 (58/79)	72.5 (95/131)	61.7 (58/94)	81.9 (95/116)	72.9 (153/210)	0.791	<0.001

Data in parentheses are raw data used to calculate percentages. AUROC = area under the receiver operator curve; DA = diagnostic accuracy = (TP + TN)/(TP + FP + TN + FN); F = fibrosis stages; FN = false negative; FP = false positive; LSN = liver surface nodularity; NPV = negative predictive value; PPV = positive predictive value; TN = true negative; TP = true positive.

### Interobserver Variability

The interobserver agreement of LSN scores from the two readers is shown in Table 5. All ICCs for interobserver agreement were higher than 0.8, indicating good reliability. According to fibrosis stages, the ICCs were 0.844 for LSN measurements in F_0_ stage, 0.801 for F_1_, 0.821 for F_2_, 0.807 for F_3_ and 0.804 for F_4_. For ROIs, the ICCs were 0.986 for ROI_1_, 0.972 for ROI_2_, 0.989 for ROI_3_, 0.988 for ROI_4_, and 0.990 for ROI_5_.

**TABLE 5:** Interobserver Variability in LSN Measurements

	Reader A	Reader B	*P* value*		Intrarater Reliability (ICC)†	95% CI		*P* value^a^
						Lower Bound	Upper Bound	
Fibrosis stage								
F0 (*n* = 29)	0.75 ± 0.04 [5]	0.74 ± 0.04 [6]	0.510		0.844	0.668	0.927	<0.001
F1 (*n* = 20)	0.83 ± 0.05 [7]	0.82 ± 0.06 [7]	0.445		0.801	0.498	0.921	<0.001
F2 (*n* = 32)	1.08 ± 0.06 [6]	1.07 ± 0.06 [5]	0.366		0.821	0.633	0.912	<0.001
F3 (*n* = 50)	1.17 ± 0.10 [9]	1.18 ± 0.11 [9]	0.743		0.807	0.659	0.890	<0.001
F4 (*n* = 79)	1.26 ± 0.10 [8]	1.26 ± 0.09 [7]	0.418		0.804	0.694	0.875	<0.001
ROIs								
ROI_1_ (*n* = 210)	1.28 ± 0.29 [23]	1.28 ± 0.27 [21]	0.324		0.986	0.981	0.989	<0.001
ROI_2_ (*n* = 210)	0.84 ± 0.22 [26]	0.83 ± 0.25 [30]	0.280		0.972	0.963	0.979	<0.001
ROI_3_ (*n* = 210)	1.12 ± 0.32 [29]	1.12 ± 0.31 [28]	0.628		0.989	0.986	0.992	<0.001
ROI_4_ (*n* = 210)	1.08 ± 0.26 [24]	1.09 ± 0.26 [24]	0.413		0.988	0.985	0.991	<0.001
ROI_5_ (*n* = 210)	1.17 ± 0.26 [22]	1.17 ± 0.25 [21]	0.147		0.990	0.987	0.993	<0.001

ICC = intraclass correlation coefficient; CI = confident interval.

LSN scores of each reader in three groups are presented as means ± SD (mean coefficient of variance, %).

* The LSN score differences of two readers were analyzed by the paired *t*‐test.

^a^ The intrarater reliability between two readers was analyzed by intraclass correlation (ICC) test.

## Discussion

This study demonstrated regional LSN analysis on a single axial MR image. The processing time using the WALTS program for a single ROI LSN measurement was reduced by 75.5% compared with the mean LSN measurement. The regional ROI_5_ LSN score demonstrated the strongest correlation with the mean LSN score and the highest DA in the differentiation between early fibrosis (≤F_2_) and advanced fibrosis (>F_2_). In the mean LSN measurement method, LSN scores showed excellent diagnostic performance in the quantification of LSN on routine liver MR scans as a part of a morphologic assessment. In prior studies, Smith et al^5^ and Pickhardt et al^6^ reported the excellent DA of mean LSN score using MRI and CT images in predicting fibrosis or cirrhosis (AUROC of 0.910 and 0.959, respectively). Our findings from mean LSN scores are well matched with previous studies.^5^, ^6^ Our results imply that both mean and regional LSN score from a single MRI image have potential as a diagnostic tool for staging hepatic fibrosis.

The reproducibility, repeatability, and DA are important in evaluating the performance of quantitative analysis.^17^ This study investigated the variability in LSN measurements, with CV values for mean LSN measurements being <10% and for single ROI_*n*_ LSN measurements being <20%. Although the variability of the single regional measurement method was approximately double that of the mean measurement method, the processing time for the single ROI measurement method was reduced by 75%. The single ROI measurement method for LSN score might therefore be helpful for clinical application. In this study, the variability in all LSN measurements was reproducible as previous MR studies.^7^, ^8^ For evaluating hepatic fibrosis, MR elastography (MRE) for assessing liver stiffness provided strong correlation with histopathologic fibrosis stage (*r* = 0.74) and high diagnostic performances for detection of stages F_2_–F_4_, F_3_–F_4_, and F_4_ (AUROCs of 0.87, 0.91, and 0.89, respectively) compared to other methods.^18^, ^19^, ^20^ Also, MRE maps showed fair to good accuracy for detection of fibrosis (AUROC range 0.76–0.84).^18^ MRI‐based fibrosis markers negatively correlated with histologic stage: contrast enhancement index (CEI) (*r* = −0.786); liver‐spleen contrast ratio (LSC) (*r* = −0.718); liver‐portal vein contrast ratio (LPC) (*r* = −0.448); and signal intensity ratio (SIR) (*r* = −0.617).^21^ For diagnosis of either significant liver fibrosis (≥F_2_) or cirrhosis (F_4_), the CEI provided better diagnostic accuracy (AUROC 0.898 and 0.881) than the aspartate aminotransferase‐to‐platelet ratio index (APRI) (AUROC 0.699 and 0.715). The CEI showed similar diagnostic accuracy for ≥F_2_ or F_4_ when using transient elastography (TE) (AUROC 0.866 and 0.884) or elastography point quantification (ElastPQ) (AUROC 0.751 and AUROC 0.786; *P* = 0.234).^21^, ^22^, ^23^, ^24^ Unlike MRE,^18^, ^19^, ^20^ imaging‐based quantification methods have the advantage that they are available in routine MRI and CT imaging in clinical practice. The methods of liver stiffness measurement, such as ultrasound elastography and MRE, also require specific devices and additional data acquisition.^18^, ^21^ The WALTS software‐based LSN measurement may provide a reproducible way to quantify fibrosis using routine MR images in clinical practice.

Several studies suggest that early stage liver fibrosis may be reversible.^25^, ^26^, ^27^, ^28^ Therefore, an accurate and easy‐implemented noninvasive diagnosis method to differentiate early and advanced liver fibrosis is important for therapeutic strategies. In this study, a single ROI_5_ LSN measurement was useful for rapidly discriminating between early (F_0_–F_2_) and advanced fibrosis groups (F_3_–F_4_).

### Limitations

First, this study was retrospective and used a nonrandomized design; therefore, enrolled patients had heterogeneous diseases including chronic hepatitis B, chronic hepatitis C, alcoholic liver disease, NALFD/NASH, and autoimmune hepatitis. Our study population predominantly included patients with chronic hepatitis B (54%) and 18% had unknown cause. Moreover, because most cases were hepatitis B viral infection, macronodular liver cirrhosis would be predominant in the liver cirrhosis (F_4_) group. Therefore, this finding might potentially lead to false‐positive or true‐negative findings due to the effect of dominant hepatitis B, and thus, the diagnostic performance might be overestimated. To overcome this shortcoming, a large‐scale multicenter study in a specific disease such as chronic hepatitis B and NAFLD should be conducted in the future. Second, there was no consideration for the effects of cross‐MRI (magnetic field strength and/or different vendors), cross‐institutional protocol, or cross‐modality reproducibility of LSN score. Thus, further study is necessary to clarify this. Finally, we focused on a regional analysis on a METAVIR classified axial‐plane MR image in the LSN measurement. We excluded patients who had undergone hepatectomy or had a pathologic condition that may affect the anterior or anterolateral surface. The axial image plane may be substituted by LSN measurement on another image‐plane (coronal or sagittal plane image) and/or another surface.

### Conclusions

This study showed regional differences in LSN scores in the liver. A single ROI measurement to evaluate the LSN score is an efficient way of saving processing time in clinical practice. The LSN score in the posterior region of the right lobe (ROI_5_) may help differentiate fibrosis stages rapidly in clinical practice. Further studies are necessary to validate whether an LSN score is a simple alternative to the mean LSN measurement score for the diagnosis of hepatic fibrosis in various liver diseases and to investigate the external validation of LSN measurement technique.

## Conflict of Interest

The authors have declared that no conflict of interest exists.

## Authors Contributions

T.H.K., Y.R.K., K.H.Y.: substantial contributions to the conception and design; T.H.K., Y.R.K., C.W.J., H.J.K., J.W.K., Y.H.L.: the acquisition, analysis, interpretation of the data; T.H.K., Y.R.K., C.W.J., H.J.K., J.W.K., Y.H.L., K.H.Y.: the drafting of the article or critical revision for important intellectual content; T.H.K., Y.R.K., K.H.Y.: final approval of the version to be published; T.H.K., K.H.Y.: agreement to be accountable for all aspects of the work in ensuring that questions related to the accuracy or integrity of any part of the article are appropriately investigated and resolved.
